# Specific Depressive Symptoms and Primary Tumor Location as Potential Predictors of Smoking Maintenance After Head and Neck Cancer Treatment

**DOI:** 10.1002/pon.70404

**Published:** 2026-02-19

**Authors:** Ana Daniela Spínola‐Silva, Jéssica Soares Bugiga, Bruna Amélia Moreira Sarafim‐Costa, Gabrielle Dias Duarte, Ana Lívia Santos‐Sousa, Rafael Akira Tzanno Murayama, Aline Satie Takamiya, Éder Ricardo Biasoli, Vitor Bonetti Valente, Glauco Issamu Miyahara, Daniel Galera Bernabé

**Affiliations:** ^1^ Psychosomatic and Education Research Center Oral Oncology Center São Paulo State University (Unesp) School of Dentistry Araçatuba São Paulo Brazil; ^2^ Department of Diagnosis and Surgery São Paulo State University (Unesp) School of Dentistry Araçatuba São Paulo Brazil

**Keywords:** anxiety, cancer, depression, head and neck, psychological factors, smoking cessation, treatment

## Abstract

**Background:**

Despite the known benefits of smoking cessation for head and neck cancer (HNC) patients, a significant proportion continue to use tobacco after treatment. Although the causes of this phenomenon are multifactorial, the underlying psychological mechanisms are still poorly understood.

**Aim:**

Investigate the influence of sociodemographic, clinicopathological, and psychological factors on smoking cessation after treatment of HNC.

**Methods:**

This study included 71 smoking HNC patients who had completed cancer treatment for at least 12 months. Clinicopathological characteristics and anxiety and depression symptoms extracted the Beck Anxiety Inventory (BAI) and Beck Depression Inventory (BDI) were evaluated in the pre‐treatment period. Data on smoking history was assessed through a semi‐structured interview.

**Results:**

A proportion of patients with HNC patients (39.4%) continued to smoke immediately after completing cancer treatment, with this proportion rising to 43.7% after 12 months of treatment. Logistic regression analyses showed that the occurrence of the primary tumor in the oral cavity (*β* = 6.891, *P* = 0.008) and the psychological symptom of sadness measured by the BDI (*β* = 5.279, *P* = 0.023) were predictive of smoking maintenance 12 months after the end of cancer treatment. Feeling like a failure before cancer treatment was the only predictor variable for smoking maintenance immediately after and 12 months after the end of treatment (*β* = 13.455, *p* < 0.001; *β* = 4.537, *P* = 0.043; respectively).

**Conclusion:**

This study presents exploratory insights that identifies pre‐treatment specific depressive symptoms and primary tumor location as promising predictive factors for continued tobacco use in patients treated for head and neck cancer.

## 1 Background

Patients with head and neck cancer who smoke at diagnosis have a significantly increased cancer‐related mortality death rate [1]. Smoking cessation is associated with a better response to oncological treatment, increased survival, and improved quality of life [2]. Smoking maintenance, especially at high intensity, can reduce the antitumor effects of radiotherapy and chemotherapy and increase postoperative complications [3, 4]. Moreover, smoking maintenance after cancer treatment also increases the rates of recurrence and second primary tumors [5, 6]. Nevertheless, a significant number of smoking patients maintain their addiction throughout cancer treatment and the follow‐up period [7].

To help cancer patients quit their addiction, it is necessary to recognize that changing smoking habits is a multifactorial and complex process, as well as to identify the main factors that may be related to the tobacco dependence [8]. HNC patients with poor social support, unmarried status, and lower social‐emotional functioning are more likely to continue smoking [9, 10]. Clinicopathological factors can also influence smoking cessation and longer abstinence periods after HNC treatment, including postoperative radiotherapy, advanced clinical stage, and tumor location [9].

Psychological factors had emerged as significant predictors of smoking in cancer patients [9, 11]. Depressive symptoms among HNC patients were associated with greater difficulty in achieving smoking cessation [12]. Furthermore, a high mental health burden was also discussed as a predictor of smoking and/or heavy drinking [12]. While previous studies have assessed psychological symptoms following HNC treatment, the relationship between pre‐treatment clinical and psychological variables and smoking maintenance after oncological treatment remains underexplored. Thus, the aim of this study is to investigate the association of sociodemographic, clinicopathological, biobehavioral, and psychological factors with smoking maintenance after HNC treatment.

## 2 Methods

This is a retrospective cohort study, approved by the Research Ethics Committee of the São Paulo State University (Unesp), School of Dentistry, Araçatuba, SP, Brazil (Protocol number: 4.425.141).

### 2.1 Patients

The study included a convenience sample of patients diagnosed with head and neck squamous cell carcinoma (HNSCC) who were treated at the Oral Oncology Center, São Paulo State University (Unesp), School of Dentistry, Araçatuba, SP, Brazil, between 2010 and 2021. The patients included in this study were selected from our database and recruited according to the following inclusion criteria: being active smokers at the time of diagnosis, having been treated for HNSCC, having pre‐treatment data available on anxiety and depression symptoms, and having completed a 1‐year post‐treatment follow‐up. Throughout the post‐treatment follow‐up period, patients were monitored by the oncology team during periodic consultations, where they received counseling on the importance of smoking cessation for preventing tumor recurrence and second primary tumors. Patients were excluded if they had undergone specific treatment for smoking cessation, used psychiatric medications, had a primary tumor located outside the oral cavity, pharynx (hypopharynx and oropharynx), or larynx, or had a cognitive deficit that precluded understand of the questionnaire.

### 2.2 Study Variables

#### 2.2.1 Sociodemographic and Clinicopathological Variables

Sociodemographic, clinicopathological, and biobehavioral (alcohol and tobacco consumption at the time of diagnosis) data were obtained from the patients' medical records. The tumor location was classified into three anatomical regions: oral cavity, pharynx, and larynx. The clinical staging of HNC was defined according to the American Joint Committee on Cancer (AJCC) and the Union for International Cancer Control (UICC) [13].

#### 2.2.2 Psychological Variables

Data regarding anxiety and depression symptoms were extracted from the database of the Psychosomatic and Education Research Center, Oral Oncology Center, São Paulo State University (Unesp), School of Dentistry, Araçatuba, SP, Brazil. These data were collected using the Beck Anxiety Inventory (BAI) [14] and Beck Depression Inventory (BDI) [15] before the initiation of oncological treatment. Both scales consist of 21 items designed to assess specific anxiety and depressive symptoms, respectively, that the respondent has experienced over the previous two weeks and on the current day. Each item is presented with four statements arranged in order of increasing severity and are scored from 0 to 3. The BAI and BDI provide a final score from 0 to 63, with higher scores indicating greater anxiety or depressive symptoms [14, 15]. The final score for the BAI was classified as minimal anxiety (0–7), mild anxiety (8–15), moderate anxiety (16–25), and severe anxiety (30–63) [14]. For the BDI, the final score was classified as minimal depression (0–9), mild depression (10–18), moderate depression (19–29), and severe depression (30–63) [15].

#### 2.2.3 Smoking Status

A structured interview was conducted with each patient to explore the trajectory of smoking throughout cancer treatment and the post‐treatment period. Patients were asked about their age at smoking initiation, whether they lived with smokers in their childhood, the type of cigarette, and the intensity of smoking at the time of diagnosis. Tobacco consumption was categorized as light use (up to 10 cigarettes per day), moderate use (11–20 cigarettes per day), and heavy use (more than 20 cigarettes per day) [16]. The smoking status of each patient was recorded retrospectively at two points. The first assessment was conducted immediately after the end of oncological treatment, and the second was conducted 12 months after the end of treatment. Each patient was asked to record their smoking status in two different periods, the first immediately after the end of cancer treatment and the second 12 months later.

### 2.3 Statistical Analysis

All statistical analyses were performed using the SPSS program (IBM SPSS Statistics version 21 Inc., Chicago, IL, USA). Chi‐square and Fisher's exact tests were used to investigate the association between the study variables and the outcomes of smoking status (immediately and 12 months after the end of cancer treatment). Missing data was handled using the pairwise deletion method. Tumor location was categorized into tumors located in the oral cavity and tumors located in other regions. Marital status was divided into married and not married. Psychological variables were analyzed using two distinct approaches. First, according to the global BAI and BDI values, patients were categorized with lower (minimal and mild) or higher (moderate and severe) levels of anxiety and depression symptoms, respectively. Second, individual items from the BAI and BDI questionnaires were analyzed as binary variables (presence or absence of each specific symptom). For this analysis, responses indicating “not applicable” were classified as absence of the symptom, while the other responses from mild to severe intensity were classified as presence of the symptom. Variables showing a *p*‐value < 0.2 in the univariate analysis for association with the response variables were included in the subsequent multivariate logistic regression analyses, which were performed using the stepwise method. The multivariate models examined sociodemographic, clinicopathological, biobehavioral, and psychological predictors of smoking maintenance versus cessation at both post‐treatment time points. The robustness of the logistic regression model was evaluated via a cross‐validation approach using k‐fold (*k* = 3). The sample was randomly divided into three subsets of approximately the same size. The model was trained on two‐thirds of the data and tested in the remaining third, repeating this process in rotation for all folds. Performance was assessed using the area under the ROC curve (AUC), sensitivity, and specificity. The level of statistical significance was set at 5% (*p* < 0.05) for all statistical tests.

## 3 Results

### 3.1 Sociodemographic, Clinicopathological, and Biobehavioral Characteristics of Smoking HNC Patients

A sample of 71 patients was included in the study. Sixty‐four of the patients were male (90.1%), and 49 patients (69%) were between 45 and 65 years old after HNC treatment (Table 1). The mean age of the participants was 61.7 years. Forty‐six patients (64.8%) were white and/or married. After HNC treatment, 60 patients (84.5%) did not live alone, while 47 (66.3%) reported being Catholic. Regarding educational level, 18 patients (25.4%) had completed middle school and 16 (22.5%) had not completed it. The monthly income of most patients was between R$1000 and R$5000 (*n* = 37; 52.1%) or lower than R$1000 (*n* = 32; 45.1%).

**TABLE 1:** Sociodemographic and clinicopathological data of smoking HNC patients.

Variable	*N* (%)
Sociodemographic variables	
Sex	
Male	64 (90.1)
Female	7 (9.9)
Age	
0–45 years	1 (1.4)
45–65 years	49 (69.0)
> 65 years	21 (29.6)
Race	
White	46 (64.8)
Non‐white	25 (35.2)
Marital status	
Married	46 (64.8)
Divorced	12 (16.9)
Single	8 (11.3)
Widowed	5 (7.0)
Living alone	
No	60 (84.5)
Yes	11 (15.5)
Religion	
Catholicism	47 (66.3)
Protestantism	12 (16.9)
Others	12 (16.8)
Education	
Incomplete elementary school	9 (12.7)
Elementary school	28 (39.3)
Middle school	25 (35.3)
High school	6 (8.5)
University education	3 (4.2)
Income	
< R$ 1.000	32 (45.1)
R$ 1.000 to R$ 5.000	37 (52.1)
> R$5.000	2 (2.8)
Medical variables	
Comorbidity	
No	46 (64.8)
Yes	25 (35.2)
Tumor location	
Oral cavity	46 (64.8)
Pharynx (hypopharynx or oropharynx)	14 (19.7)
Larynx	11 (15.5)
Clinical stage	
Early (I/II)	46 (64.8)
Advanced (III/IV)	25 (35.2)
Treatment	
Surgery	36 (50.8)
Surgery + radiotherapy	15 (21.1)
Radiotherapy	10 (14.1)
Radiotherapy + chemotherapy	5 (7.0)
Surgery + radiotherapy + chemotherapy	5 (7.0)
Biobehavioral data	
Alcohol consumption^a^	
None	8 (11.3)
Current drinkers	38 (53.5)
Former drinkers	25 (35.2)
Type of cigarette^a^	
Straw cigarette	2 (2.8)
Paper cigarette	49 (69.0)
Straw and paper cigarette	20 (28.2)
Intensity of tobacco use^a^	
Light	8 (11.3)
Moderate	28 (39.4)
Severe	35 (49.3)
Age of first cigarette use	
Childhood (0–11 years)	15 (21.1)
Adolescence (12–18 years)	38 (53.5)
Adulthood (> 18 years)	18 (25.4)
Living with a smoker in childhood	
No	10 (14.1)
Yes	61 (85.9)
Time of smoking	
20–30 years	8 (11.3)
30–40 years	18 (25.4)
40–50 years	29 (40.8)
> 50 years	16 (22.5)
Tobacco consumption	
After the end of treatment	
No	43 (60.6)
Yes	28 (39.4)
12 months after the end of treatment	
No	40 (56.3)
Yes	31 (43.7)

*Note: N* = number of patients; *y* = years.

^a^ At the time of diagnosis.

Forty‐six HNC patients (64.8%) had no comorbidities, while 25 (35.2%) reported at least one other disease besides cancer (Table 1). In the current study, 46 patients (64.8%) had a tumor in the oral cavity, 14 (19.7%) in the pharynx (hypopharynx or oropharynx), and 11 (15.5%) in the larynx. Forty‐six patients (64.8%) were diagnosed with early‐stage disease (stage I or II), while 25 (35.2%) had cancer at an advanced stage (III or IV). Regarding the modality of cancer treatment, 36 patients (50.8%) underwent only surgery, 15 (21.1%) underwent surgery and radiotherapy, and 10 (14.1%) underwent radiotherapy only, while 5 patients (7%) received radiotherapy and chemotherapy or all three types of treatment (Table 1).

Thirty‐eight patients (53.3%) were current drinkers, 25 (35.2%) were former drinkers, and only 8 (11.3%) had no history of alcohol consumption at the time of HNC diagnosis (Table 1). All patients were smokers when they noticed the first sign or symptom of the disease. Paper cigarettes were the main type of cigarette consumed by most individuals (*n* = 49; 69%). Thirty‐five patients (49.3%) consumed tobacco heavily, 28 (39.4%) moderately, and 8 (11.3%) lightly. In this study, 38 patients (53.5%) started cigarette smoking during adolescence (between 12 and 18 years old), 18 (25.4%) in adulthood (over 18 years old), and 15 (21.1%) during childhood (under 12 years old). Sixty‐one patients (85.9%) reported that, during their childhood, they lived with a relative who was a smoker. The average duration of smoking habits in the study sample was 43.1 years. Twenty‐nine patients (40.8%) smoked for between 40 and 50 years, 18 (25.4%) between 30 and 40 years, and 16 (22.5%) for over 50 years. Regarding tobacco consumption, 39.4% of the patients continued to smoke immediately after completing cancer treatment, with this proportion rising to 43.7% after 12 months of treatment (Table 1).

### 3.2 Anxiety and Depression Symptoms in Smoking HNC Patients

Anxiety and depression symptoms were evaluated in smoking HNC patients before starting treatment (Table 2). One patient did not report data regarding the levels of anxiety symptoms, and three patients did not provide data on their levels of depressive symptoms. The BAI self‐report scale identified that 63 patients (88.7%) had at least one anxiety symptom. Forty‐nine patients (70%) had minimal, 18 (25.7%) mild, and 2 (2.9%) moderate symptoms. One patient (1.4%) displayed severe anxiety symptoms. Depression symptoms were reported by 54 individuals (76.1%), according to the BDI self‐report scale. Fifty‐one patients had minimal (75%), 11 (16.2%) mild, and 5 (7.3%) moderate symptoms. Only one (1.5%) patient showed severe depressive symptoms.

**TABLE 2:** Anxiety and depression symptoms in smoking HNC patients.

Variables	*N* (%)
Anxiety symptoms^a^ ^,^ ^c^	
Present	63 (90.0)
Absent	7 (10.0)
Level of anxiety symptoms	
Minimum	49 (70)
Mild	18 (25.7)
Moderate	2 (2.9)
Severe	1 (1.4)
Depression symptoms^b^	
Present	54 (76.1)
Absent	14 (19.7)
Unreported data	3 (4.2)
Level of depression symptoms	
Minimum	51 (75)
Mild	11 (16.2)
Moderate	5 (7.3)
Severe	1 (1.5)

*Note: N* = number of patients.

^a^ BAI self‐reported scale.

^b^ BDI self‐reported scale.

^c^ Variable with missing data.

### 3.3 Marital Status, Tumor Location, and Time of Smoking Are Associated With Quitting Smoking Outcomes After HNC Treatment

The univariate analysis showed that divorced patients with HNC had greater difficulty quitting smoking compared to single, married, and widowed patients, both immediately (*p* = 0.015) and 12 months (*p* = 0.031) after the end of oncological treatment (Table 3). This same difficulty was observed in patients with tumors located in the oral cavity. These patients had more difficulty quitting smoking compared to those with tumors located in the pharynx or larynx (*p* < 0.05). Patients who smoked for 40–50 years had greater difficulty quitting smoking compared to patients who smoked for less than 40 years, both immediately (*p* = 0.005) and 12 months after the end of treatment (*p* = 0.014).

**TABLE 3:** Associations between variables and quitting smoking outcomes after HNC treatment.

Variables	Smoking maintenance	
	Immediately after cancer treatment	12 months after cancer treatment
Divorced marital status	0.015^a^ ^,^ ^f^	0.031^a^ ^,^ ^f^
Tumor location in oral cavity	0.047^b^ ^,^ ^f^	0.012^b^ ^,^ ^f^
Time of smoking (40–50 years)	0.005^c^ ^,^ ^f^	0.014^c^ ^,^ ^f^
Anxiety symptoms		
Higher levels of anxiety symptoms	0.022^d^ ^,^ ^f^	0.004^e^ ^,^ ^f^
Tremors in the legs	0.044^d^ ^,^ ^f^	0.079^d^
Unable to relax	0.044^d^ ^,^ ^f^	0.017^d^ ^,^ ^f^
Imbalance	0.025^d^ ^,^ ^f^	0.068^d^
Nervous	0.020^d^ ^,^ ^f^	0.064^d^
Depression symptoms		
Higher occurrence of depression symptoms	0.022^e^ ^,^ ^f^	0.043^e^ ^,^ ^f^
Sadness	0.002^d^ ^,^ ^f^	0.011^d^ ^,^ ^f^
Feeling like a failure	0.002^d^ ^,^ ^f^	0.023^d^ ^,^ ^f^
Guilty	0.008^d^ ^,^ ^f^	0.008^d^ ^,^ ^f^
Feeling worse than others	0.036^d^ ^,^ ^f^	0.347^d^
Suicidal thoughts	0.029^d^ ^,^ ^f^	0.046^d^ ^,^ ^f^
Difficulty making decisions	0.034^d^ ^,^ ^f^	0.350^d^
Difficulty sleeping	0.022^d^ ^,^ ^f^	0.091^d^
No health concerns	0.035^d^ ^,^ ^f^	0.077^d^

^a^ Values were measured with the categories married, divorced, single or widowed.

^b^ Values were measured with the categories oral cavity, pharynx and larynx.

^c^ Values were measured with the categories < 20 years, 20–30 years, 30–40 years, 40–50 years, > 50 years.

^d^ Values were measured with the severity categories (minimum, mild, moderate, or severe).

^e^ Values were measured with the intensity measure (lower or higher).

^f^ *p* < 0.05 (Chi‐square test or Fisher's exact test).

### 3.4 Psychological Symptoms in Pre‐Treatment are Related to Quitting Smoking Outcomes After HNC Treatment

Pre‐treatment lower levels of anxiety symptoms were associated with smoking cessation both immediately (*p* = 0.022) and 12 months (*p* = 0.004) after the end of treatment (Table 3). Patients who reported tremors in their legs and difficulty relaxing were more likely to continue smoking after HNC treatment (*p* < 0.05). Those who reported imbalance (*p* = 0.025) and being very nervous (*p* = 0.020) before starting HNC treatment were more likely to maintain smoking addiction immediately after the end of treatment. The higher occurrence of depressive symptoms as measured by the BDI was associated with smoking maintenance both immediately (*p* = 0.022) and 12 months after completing the treatment (*p* = 0.043). Patients who reported feeling sadness, feeling like a failure, or guilty before starting cancer treatment were more likely to continue tobacco consumption immediately and 12 months after the end of cancer treatment (*p* < 0.05). According to the univariate analysis, depressive symptoms such as feeling worse than others (*p* = 0.036), having suicidal thoughts (*p* = 0.029), difficulty making decisions (*p* = 0.034) or sleeping (*p* = 0.022), and not having health concerns (*p* = 0.035) were related to the maintenance of tobacco consumption after cancer treatment.

### 3.5 Tumor Location and Depression Symptoms May Predict the Risk of Smoking Maintenance After HNC Treatment

In the multivariate analysis, HNC patients with primary tumors located in the oral cavity were more likely to continue smoking 12 months after cancer treatment compared to patients who had tumors in the pharynx or larynx (*β* = 6.891, 95% CI = 1.661–28.580, *p* = 0.008; Table 4). Patients who reported feeling like a failure had greater difficulty in quitting smoking soon (*β* = 13.455, 95% CI = 3.210–56.395, *p* < 0.001) and 12 months after cancer treatment (*β* = 4.537, 95% CI = 1.049–19.628, *p* = 0.043) compared to patients who did not report such feelings in the BDI interview before cancer treatment. Patients who reported feeling sad during the pre‐treatment period were 5.2 times more likely to continue smoking 12 months after the end of treatment compared to patients who did not feel sadness (*β* = 5.279, 95% CI = 1.254–22.235, *p* = 0.023). To assess the robustness of the stepwise logistic regression model, the area under the ROC curve (AUC) was calculated using k‐fold cross‐validation with *k* = 3. For the smoking maintenance immediately after the oncological treatment outcome, in the internal validation via k‐fold (*k* = 3), an AUC of 0.866 was observed in Group 1, with a sensitivity of 71.4% and a specificity of 100% at the optimal cutoff point. The other groups showed variable performance, suggesting limitations in the model's generalizability for this outcome. For the outcome of smoking maintenance 12 months post‐treatment the cross‐validation yielded a mean AUC of 0.822 (range: 0.734–0.913), with a mean sensitivity and specificity of 96.9% and 49.8%, respectively. These findings demonstrate that the model for the outcome of smoking maintenance 12 months after the end of oncological treatment exhibit robust and consistent discriminatory performance across the different subsets of the sample.

**TABLE 4:** Predictors of smoking maintenance after HNC treatment.

Independent variable	Smoking maintenance					
	After cancer treatment			12 months after cancer treatment		
	OR	95% CI	*p* value	OR	95% CI	*p* value
Tumor location (oral cavity)^a^ ^,^ ^b^	—	—	—	6.891	1.661–28.580	0.008^d^
Feeling like a failures^a^ ^,^ ^b^	13.455	3.210–56.395	< 0.001^d^	4.537	1.049–19.628	0.043^d^
Sadness^a^ ^,^ ^c^	—	—	—	5.279	1.254–22.235	0.023^d^

^a^ These variables remained in the final regression model.

^b^ Values were measured with the categories (tumor located in the oral cavity and tumor in pharynx or larynx).

^c^ Values were measured with the binary measure (yes or no).

^d^ *p* < 0.05.

## 4 Discussion

The results of the current study showed that almost half of the patients continued to smoke one year after completing HNC treatment. Most patients exhibited anxiety and depressive symptoms at the time of diagnosis, and some psychological variables were associated with smoking cessation outcomes after cancer treatment. Patients who felt sad before starting HNC treatment were more likely to maintain smoking habits 12 months after the end of oncological treatment compared to those who did not report this feeling. Similarly, after adjusting for potential confounding variables, HNC patients who reported feeling like a failure before treatment onset or had a tumor located in the oral region were more likely to continue smoking both immediately and 12 months after the end of treatment. This study provides exploratory insights that identify promising predictive factors to understanding the smoking maintenance among HNC patients.

According to the multivariate analysis, the results demonstrated that patients with primary tumors in the oral cavity were 6.8 times more likely to continue smoking compared to patients with primary neoplasms in other locations. Along with this study's results, Ostroff et al. showed that patients with tumors located in the oral cavity were 3.2 times more likely to continue smoking than individuals with tumors in the larynx or pharynx [17]. The reasons for this finding are not easily identifiable. In comparison with tumors located in oropharyngeal, hypopharyngeal, and pharynx, primary lesions located in the oral cavity undergo more conservative treatments and presents lower functional sequelae after oncological treatment [18, 19]. We hypothesize that reduced complications associated with oral cancer treatment may lead patients to underestimate the severity of the cancer and its etiological factors, which could negatively influence the patient's decision to quit smoking. In contrast, laryngeal and oropharyngeal tumors are less visible due to their anatomical localization and are likely to be advanced at the time of diagnosis, requiring more aggressive treatment [20]. The severity of the disease and its treatment can significantly influence patients' decision to quit smoking [21, 22]. Multicenter studies with larger sample sizes would strengthen these findings and clarify the potential relationship between primary tumor location and smoking maintenance in HNC patients.

Beyond clinicopathological variables, the associations between anxiety and depression symptoms and the maintenance of smoking habits in HNC patients have been widely explored [9, 23]. However, there is a lack of research on specific psychological symptoms that may contribute to resistance to smoking cessation in HNC patients. An 18‐month observational study demonstrated that patients with different types of cancer with higher occurrence of depressive symptoms after cancer treatment were also more likely to continue smoking [24]. Nevertheless, the influence of specific depressive symptoms, as evaluated by a validated instrument, such as the BDI self‐report scale, over the smoking cessation outcomes has not yet been investigated in HNC patients. In the present study, sadness was identified as a predictive factor associated with smoking maintenance in HNC patients. Negative emotions, especially sadness, can lead to a loss of self‐control and influence reward‐seeking behavior, making quitting smoking more challenging [25, 26]. Dorison and colleagues (2020) investigated the effects of sadness, but not other negative emotions, on smoking among the general population. They concluded that sadness has a strong association with smoking status and is related to smoking relapses, cigarette cravings and increased volume of cigarette puffs [26]. Even after 20 years of abstinence, sadness has been identified as an emotional factor associated with relapses into addiction [26].

In the current study, feeling like a failure was also identified as a predictive variable for smoking maintenance 12 months after the end of cancer treatment. Low self‐esteem is influenced by patients' perceptions of how others view them and have been associated with negative self‐belief, including feelings of failure and negative self‐appraisal [27, 28]. Feeling like a failure, particularly after unsuccessful attempts to quit smoking, is linked to increased stress, psychological distress, and lower self‐efficacy, which can negatively affect motivation and success in smoking cessation efforts [29]. Feelings of frustration have been reported as an important predictor of relapses in patients who quit smoking [30]. According to a previous study by our team, based on smokers' own perceptions, negative emotions are a motivation factor for cigarette use [31]. Nonetheless, when asked by a healthcare professional, many patients do not accurately report their smoking status, often due to stigma or social unacceptability. Previous studies have indicated that 11%–33% of patients misreport their smoking status when self‐reported data is compared with biochemical analysis [32, 33, 34]. Moreover, Stuber and Galea (2009) reported that 8% of the current smokers interviewed confessed they kept their smoking status a secret from a doctor or another health care provider [35]. In the current study, however, the patient's smoking status was assessed only through self‐report data collection. Although the multivariate analysis controlled for confounding variables, the lack of biochemical confirmation narrows the interpretative scope of our findings. Future studies would benefit from biochemical validation methods to assess the patients' smoking status.

Smoking during cancer treatment can lead to negative consequences for patients with addiction [36, 37]. In the current study, 39.4% of patients were still smoking shortly after the end of treatment. This proportion increased 1 year after cancer the completion of cancer treatment. Smoking maintenance during HNC treatment can exacerbate radiotherapy toxicities, reduce overall survival, and increase the risk of a second primary tumor or disease recurrence [38]. These findings highlight the importance of smoking cessation interventions to encourage patients to overcome their addiction. However, the psychological underpinnings of smoking demonstrate the complexity and depth of roots that these habits have in patients' lives. Therefore, smoking cessation efforts in HNC patients must address the patient's mosaic of feelings, traumas, and insecurities. Most professionals address the issue of smoking cessation in cancer patients only during initial consultations and do not discuss treatment or support options for overcoming addiction [39, 40]. Smoking cessation approaches in HNC patients must consider the biological and psychological implications of smoking habits. Nicotine withdraw can exacerbate depressive symptoms and induce high stress levels in the patient. Moreover, in HNC patients, stress relief is often reported as a perceived benefit of continuing to smoke [39]. Respectful and specialized support throughout cancer treatment is essential for the success of smoking cessation efforts [8, 40, 41]. This support must include a comprehensive psychological approach, focusing on stress management and depressive symptoms experienced by HNC patients, particularly sadness and feelings of failure.

### 4.1 Study Limitations

This study has some limitations. First, the assessment of tobacco use after HNC treatment was conducted through patient interviews rather than by measuring systemic levels of nicotine metabolites. This may lead to an underreporting of smoking status, as some patients may not feel comfortable disclosing continued tobacco dependence. Second, psychological symptoms were assessed only during the pre‐treatment period and not throughout the cancer treatment. Therefore, it is not possible to determine whether levels of depression and anxiety after cancer treatment would also be influencing factors for the maintenance of tobacco dependence in the sample studied. Additionally, this study utilized a relatively small convenience sample from a single center, which limits the generalizability of the findings. The small sample size also may have contributed to the wide 95% confidence intervals observed in some results from the multivariate analyses. Finally, some data related to tobacco consumption during cancer treatment were collected retrospectively, which may be affected by the patients' memory limitations.

### 4.2 Clinical Implications

The psychological processes behind the maintenance of cigarette consumption in smoking patients after cancer treatment are still poorly understood. This study's results suggest that specific depressive symptoms before cancer treatment, such as sadness and feeling like a failure, may be associated with continued smoking in patients treated for head and neck cancer (HNC). However, these are preliminary findings with a small sample size from a single center. Despite the attractivity of these results, the relatively wide confidence intervals suggest that they should be interpretated with caution. This study provides preliminary insights into the association between psychological factors and smoking maintenance among HNC patients. Although preliminary, the results underscore the importance of developing personalized psychotherapeutic strategies to improve HNC patient care. Future multicentered research with larger sample sizes and biochemical confirmation of smoking status would help to strengthen these findings.

### 4.3 Conclusions

Our study suggests that specific pre‐treatment emotional symptoms and primary tumor location are associated with continued tobacco use after HNC treatment. Specifically, patients who reported having failed throughout their lives or had greater sadness before starting cancer treatment appeared to have more difficulty quitting smoking after treatment. This study provides new insights into smoking management in HNC patients and reinforces the potential benefits of initiating psychotherapy even before oncological treatment begins. Therefore, a deeper understanding of the relationship between pre‐treatment negative emotions and smoking habits in HNC patients could enhance the success of smoking cessation interventions and, consequently, improve cancer treatment outcomes. We hypothesize that this knowledge will assist oncology care teams in managing tobacco use among patients undergoing HNC treatment, potentially reducing rates of cessation failure. However, these are preliminary results and must be interpreted within the context of the study's limitations. Further studies are needed to deepen the understanding of the role of specific depressive symptoms in smoking maintenance among cancer patients.

## Author Contributions

**Ana Daniela Spínola–Silva:** data curation, formal analysis, investigation, methodology, writing – original draft. **Jéssica Soares Bugiga:** investigation, methodology, data curation. **Bruna Amélia Moreira Serafim–Costa:** methodology, data curation, investigation. **Gabrielle Dias Duarte:** methodology, data curation. **Ana Lívia Santos–Sousa:** writing – original draft. **Rafael Akira Tzanno Murayama:** writing – review and editing. **Aline Satie Takamiya:** writing – review and editing. **Éder Ricardo Biasoli:** writing – review and editing. **Vitor Bonetti Valente:** writing – review and editing. **Glauco Issamu Miyahara:** writing – review and editing. **Daniel Galera Bernabé:** conceptualization, data curation, formal analysis, funding acquisition, investigation, project administration, resources, supervision, validation, writing – review and editing.

## Funding

The authors have nothing to report.

## Ethics Statement

All procedures performed in studies involving human participants were in accordance with the ethical standards of the institutional and/or national research committee and with the 1964 Helsinki Declaration and its later amendments or comparable ethical standards. The study was approved by the Research Ethics Committee of the São Paulo State University (Unesp), School of Dentistry, Araçatuba, SP, Brazil (Number: 4.425.141).

## Conflicts of Interest

The authors declare no conflicts of interest.

## Data Availability

The data that support the findings of this study are available on request from the corresponding author DGB.
